# Supplementary material for: Toxin Dynamics among Populations of the Bioluminescent HAB Species Pyrodinium bahamense from the Indian River Lagoon, FL
Source: Mar Drugs. 2024 Jul 4;22(7):311. doi: 10.3390/md22070311 (PMC11277979; doi:10.3390/md22070311)
Supplement: Supplementary file 1 [file marinedrugs-22-00311-s001.zip › marinedrugs-3060054-supplementary.pdf]

Supporting Information for Cusick et al

SI Text

Results

2.1 Cell Presence and Abundance

Both our data and HAB monitoring by government agencies (HAB reports by Florida Wildlife Commission, available upon request) demonstrate the fluctuations in P. bahamense presence and abundance throughout the IRL. The overall average cell abundance (as cells L^{-1}) at each of the four sites, when assimilating all dates over the course of a year were: DB, 32,167 (±70,829); HC, 17,238 (±23,950); KP 19,865 (±16,014); B42, 26,386 (±56,661) (FIGURE 1A). Not surprisingly, there was no statistically significant difference between sites when including all dates, likely due to the high standard deviations that result from the variation in cell abundance from early in the spring to late Fall. Averaging just the samples from May, when sampling in B42 was initiated, until October, also showed no significant differences (FIGURE S1). Additionally, there were dates on which sampling occurred but P. bahamense was not detected. For example, sampling in early/mid March at DB, P. bahamense was not detected on 3-15-22, but was recorded on 3-18 at low concentrations (133 cells/L). At HC, P. bahamense was not detected on 3-16 or 3-30, but found at 53 cells/L on 3-29-22. (These dates, which would have resulted in a cell abundance of zero, were not included when calculating average cell abundance throughout the course of the season.)
Sampling exclusively at DB over consecutive days in 2023, the average cell abundance was: Mar, 709 (±815); April, 14,448 (±7081); June, 23,1520 (±199,667); July, 13570 (±11,336).

SI Figures

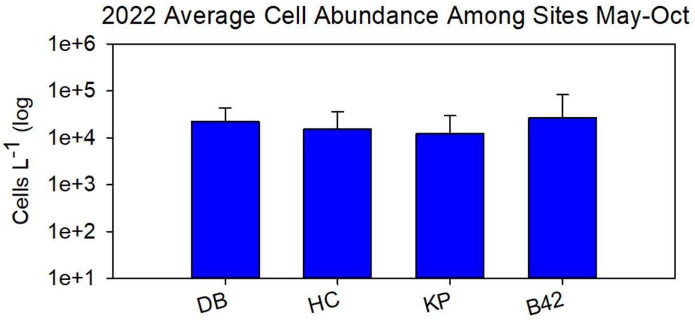

Figure S1. Average cell abundance from May (when B42 sampling started) – Oct 2022.

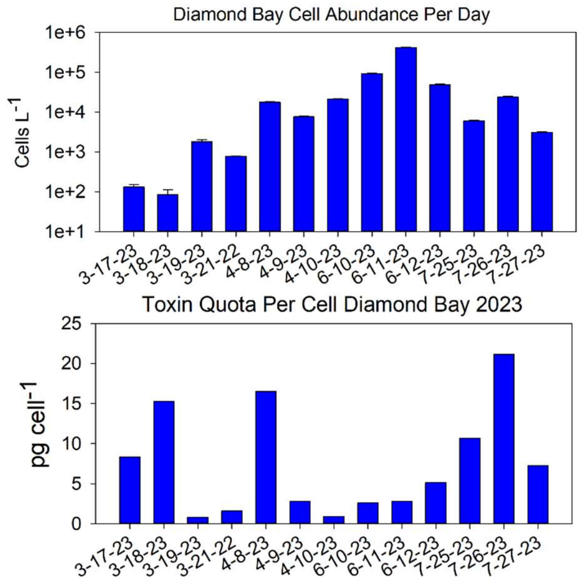

Figure S2. Profile of cell abundance and toxin quota per cell at DB on daily basis in 2023.

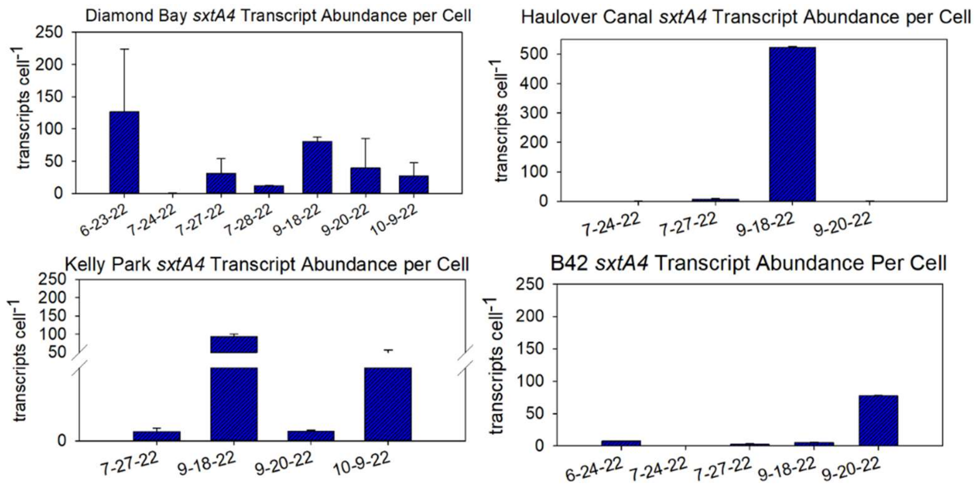

Fuigure S3. SxtA4 transcripts among the four sites in 2022.

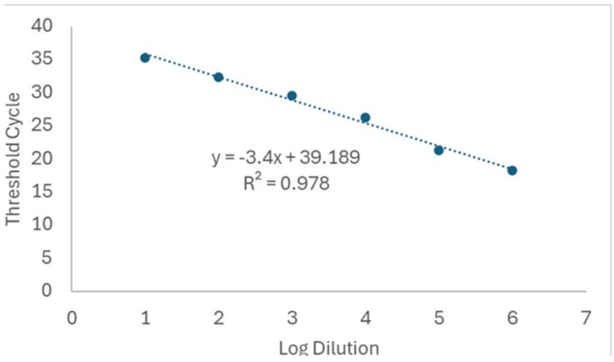

Figure S4. Standard curve of *P. bahamense rbcL* qPCR assay. Ten-fold serial dilutions were prepared using a PCR-purified nearly full-length *rbcL* gene product. The assay characteristics were: slope = -3.4, y-intercept = 39.189, r^2 = 0.978, and PCR efficiency = 96.42%,

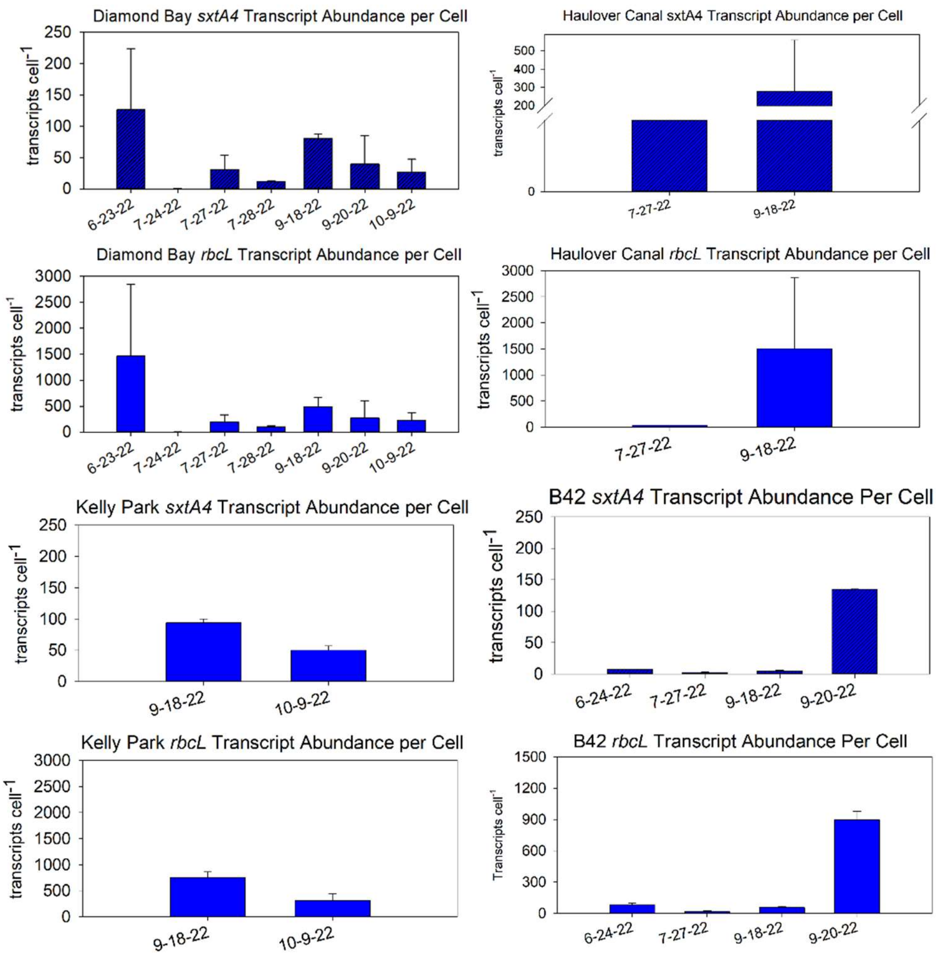

Figure S5. *SxtA4* and *rbcL* transcription show similar patterns of regulation from the four sites in 2022.

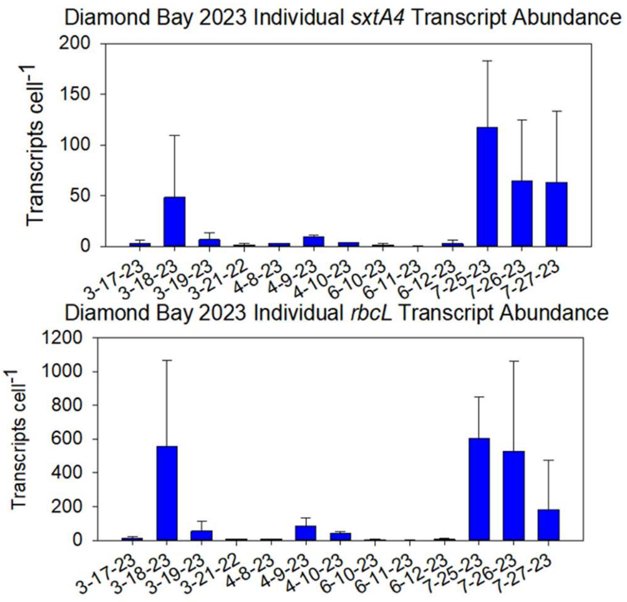

Figure S6. *SxtA4* and *rbcL* transcriptional profiles from DB in 2023.
